# Physician code creep after the initiation of outpatient volume control program and implications for appropriate ICD-10-CM coding

**DOI:** 10.1186/s12913-020-5001-5

**Published:** 2020-02-19

**Authors:** Fu-Wen Liang, Liang-Yi Wang, Lin-Yi Liu, Chung Yi Li, Tsung-Hsueh Lu

**Affiliations:** 1 Department of Public Health, College of Health Sciences, Kaohsiung Medical University, Kaohsiung, Taiwan; 2 Department of Public Health, College of Medicine, National Cheng Kung University, No. 1, Dah Hsueh Road, Tainan City, 701 Taiwan; 3 Division of Medical Affairs, National Health Insurance Administration, Taipei, Taiwan; 4 Department of Healthcare Administration, College of Medical and Health Science, Asia University, Taichung, Taiwan; 5 Department of Public Health, College of Public Health, China Medical University, Taichung, Taiwan

**Keywords:** Claims, Administrative data, Methodology, Diagnosis codes

## Abstract

**Background:**

Most studies on the physician code creep (i.e., changes in case mix record-keeping practices to improve reimbursement) have focused on episodes (inpatient hospitalizations or outpatient procedures). Little is known regarding changes in diagnostic coding practices for better reimbursement among a fixed cohort of patients with chronic diseases.

**Methods:**

To examine whether physicians in tertiary medical centers changed their coding practices after the initiation of the Outpatient Volume Control Program (OVCP) in Taiwan, we conducted a retrospective observational study of four patient cohorts (two interventions and two controls) from January 2016 to September 2017 in Taiwan. The main outcomes were the number of outpatient visits with four coding practices: 1) OVCP monitoring code recorded as primary diagnosis; 2) OVCP monitoring code recorded as secondary diagnosis; 3) non-OVCP monitoring code recorded as primary diagnosis; 4) non-OVCP monitoring code recorded as secondary diagnosis.

**Results:**

The percentage change of the number of visits with coding practice 1 between 2016Q1 and 2017Q3 was − 74% for patients with hypertension and − 73% with diabetes in tertiary medical centers and − 23% and − 17% in clinics, respectively. By contrast, the percentage changes of coding practice 3 were + 73% for patients with hypertension and + 46% for patients with diabetes in tertiary medical centers and − 19% and − 2% in clinics, respectively.

**Conclusions:**

Physician code creep occurred after the initiation of the OVCP. Education regarding appropriate outpatient coding for physicians will be relatively effective when proper coding is related to reimbursement.

## Background

In 1989, Simborg published a paper titled “DRG creep: a new hospital acquired disease.” He defined the DRG (diagnostic related group) creep as a deliberate and systematic shift in a hospital’s reported case mix to improve reimbursement [1]. Early studies have specifically focused on DRG creep and revealed that this acquired disease existed in numerous hospitals [2–6]. Subsequent studies have suggested that various types of physician code creep behaviors occurred after the introduction of value-based programs [7–18]. However, most studies on physician code creep have focused on episodes (outpatient procedures or inpatient hospitalizations) in which it was difficult to evaluate whether the increase in the use of more severe codes was due to an increase in the severity of the patient’s case or to a change in the physician’s coding behavior per se. Little is known on the changes in diagnostic coding practices for better reimbursement among a fixed cohort of patients with chronic diseases after the introduction of a value-based program within a short period.

Under the Taiwan National Health Insurance (NHI) program, the insured have the option of seeking care at any clinical level, which results in a high volume of outpatient visits at tertiary medical centers [19]. To avoid the overuse and waste of specialized resources in tertiary medical centers, an Outpatient Volume Control Program (OVCP) was proposed by the Taiwan NHI Administration in November 2016 and finalized and launched in May 2017. The OVCP stipulates that physicians in tertiary medical centers should not see too many patients with minor diseases (such as a common cold or superficial wound) or stable chronic diseases without complication and requires that physicians in tertiary medical centers transfer these patients to community hospitals or local clinics. The OVCP will monitor the volume of outpatient visits with 172 designated diagnoses (mainly minor conditions or stable chronic conditions without complication) as primary diagnosis in tertiary medical centers. If the volume of outpatient visits with these monitoring diagnosis did not decrease by 10% compared with the previous year’s volume, the NHI would not reimburse the outpatient claims that exceeded the expected volume. The initiation of OVCP provides an opportunity to examine whether physicians in tertiary medical centers changed their coding practices to avoid a reduction of reimbursement.

## Methods

### Design, settings, and participants

This is a nationwide population-based before-and-after observational study using the Taiwan NHI outpatient claims data from January 2016 to September 2017. We identified four patient cohorts for this study. The first and second patient cohorts involved patients with hypertension who visited the same physician in tertiary medical centers (cohort 1_HT_TMC) and in clinics (cohort 2_HT_Clin), respectively. The third and fourth patient cohorts included patients with diabetes mellitus who visited the same physician in tertiary medical centers (cohort 3_DM_TMC) and in clinics (cohort 4_DM_Clin), respectively. The cohort 1_HT_TMC and cohort 3_DM_TMC represented the intervention groups in which the OVCP applied, and the cohort 2_HT_Clin and cohort 4_DM_Clin were the control groups in which the OVCP would not been applied.

### Main outcome: coding practices

In Taiwan, physicians are in charge of determining the International Classification of Disease (ICD) codes for outpatient claims. The ICD Tenth Revision Clinical Modification (ICD-10-CM) was introduced on January 1st, 2016 in Taiwan. A physician can assign one ICD-10-CM code as the primary diagnosis and up to four ICD-10-CM codes as secondary diagnoses in the outpatient claims.

The OVCP would monitor the volume of outpatient visits with 172 designated diagnoses as the primary diagnosis in claims data. Most of the OVCP monitoring diagnoses are minor conditions (such as common cold, gastroenteritis, diarrhea, dizziness, or superficial wounds) are episodes, it is difficult to determine whether the increases or decreases of these code occurrences were due to changes in the incidence or coding practice. To solve this problem, we confined our observations on the number of visits with two OVCP monitoring diagnoses (i.e., essential hypertension [ICD-10-CM code I10] and diabetes mellitus without complication [ICD-10-CM code E119]). To examine the changes in physicians’ coding practices, we further examined the two related non-OVCP monitoring diagnoses, namely ICD-10-CM codes I11 − I13 “hypertensive heart or/and renal disease” and ICD-10-CM codes E110 − E118 “diabetes mellitus with complications.” The main outcome of this study was the number of visits for the following four coding practices in four patient cohorts:

- Coding practice 1: OVCP monitoring code (ICD-10-CM codes I10 or E119) recorded as primary diagnosis;
- Coding practice 2: OVCP monitoring code (ICD-10-CM codes I10 or E119) recorded as secondary diagnosis;
- Coding practice 3: non-OVCP monitoring codes (ICD-10-CM codes I11 − I13 or E110 − E118) recorded as primary diagnosis;
- Coding practice 4: non-OVCP monitoring codes (ICD-10-CM codes I11 − I13 or E110 − E118) recorded as secondary diagnoses.

To reduce the outpatient visits with OVCP monitoring diagnosis, physicians in tertiary medical centers (intervention group) might be less inclined to record OVCP monitoring codes (ICD-10-CM codes I10 or E119) as primary diagnoses, either by moving OVCP monitoring codes from primary diagnosis to secondary diagnosis or by replacing non-OVCP monitoring codes (ICD-10-CM codes I11 − I13 or E110 − E118) as primary diagnoses. Therefore, we hypothesized that the number of visits with coding practice 1 would decline; coding practices 2 and 3 would increase; and coding practice 4 would not change prominently in tertiary medical centers after the initiation of the OVCP. However, in clinics (control group), none of the aforementioned changes would be noted.

### Statistical analysis

We first calculated the number of visits based on the aforementioned four coding practices for the Q1, Q2, Q3, and Q4 of 2016 and Q1, Q2, and Q3 of 2017 in both tertiary medical centers (intervention group) and clinics (control group). We did not include the 2017 Q4 data because of their incompleteness; several outpatient claims submissions were delayed in 2018 and were not available in this study. Because the absolute number of visits in each coding practice varied considerably, we used an index change (using the number of visits for 2016Q1 as reference) to contrast the changes between those visits in the intervention group and those in the control group. The OVCP was proposed in November 2016 and finalized in May 2017 after several negotiations on the list of diagnoses for monitoring in tertiary medical centers. We thus compared the percentage of change of the number of visits for four coding practices between 2016Q1 and 2017Q3 in both the intervention and the control groups.

## Results

The four patient cohorts initially included 738,834 and 1,207,513 patients with hypertension in tertiary medical centers and in clinics, respectively; these cohorts included 452,851 and 504,482 patients with diabetes in tertiary medical centers and in clinics, respectively. The corresponding loss follow-up rates were 15.5, 24.6, 18.8, and 24.4% in 2017Q3. That is, the loss follow-up rates were higher in clinics than in tertiary medical centers.

**Fig. 1:**
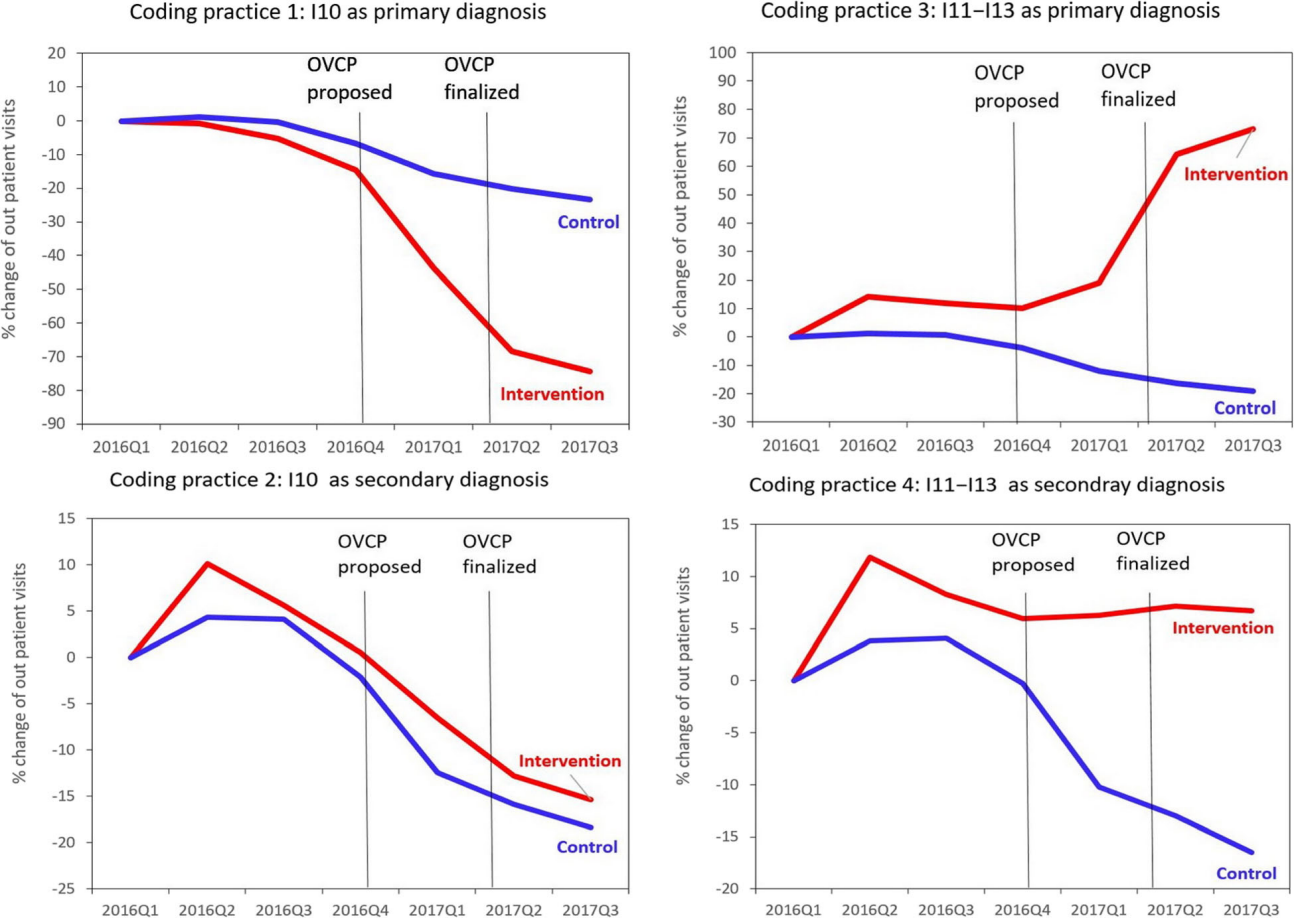
Index percent (%) change of the quarterly number of visits by different coding practices among a fix patient cohort in tertiary medical centers (intervention group) and clinics (control group) before and after the proposal and implementation of the Outpatient Volume Control Program (OVCP) in Taiwan (ICD-10-CM code I10 “essential hypertension” was a OVCP monitoring code; I11 − I13 “hypertensive heart or/and renal disease” were non-OVCP monitoring codes)

**Fig. 2:**
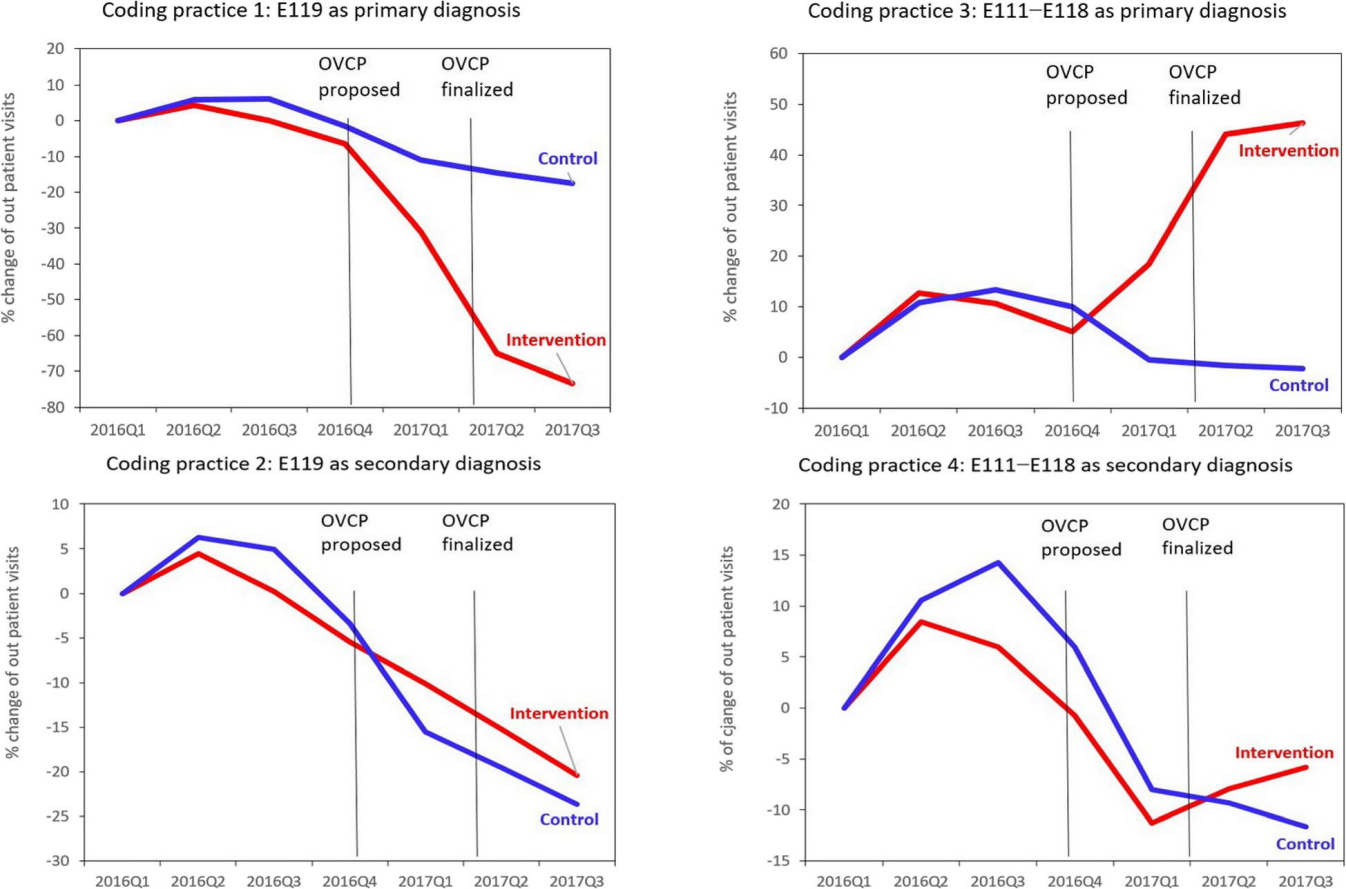
Index percent (%) change of the quarterly number of visits by different coding practices among a fix patient cohort in tertiary medical centers (intervention group) and clinics (control group) before and after the proposal and implementation of the Outpatient Volume Control Program (OVCP) in Taiwan (ICD-10-CM code E119 “diabetes mellitus without complication” was a OVCP monitoring code; E110 − E118 “diabetes mellitus with complication” were non-OVCP monitoring codes)

The numbers and indexes of outpatient visits for the four coding practices from 2016Q1 to 2017Q3 are illustrated in Table 1 and Fig. 1 for patients with hypertension and in Table 2 and Fig. 2 for patients with diabetes.

**Table 1:** Number (% change^a^) of outpatient visits (× 1000) of four hypertension-related coding practices in outpatient claims by the same physician in tertiary medical centers (intervention) and clinics (control group) from 2016Q1 to 2017Q3 in Taiwan (ICD-10-CM code I10 is the Outpatient Volume Control Program (OVCP) monitoring code and I11-I13 are non-OVCP monitoring codes)

	2016Q1		2016Q2		2016Q3		2016Q4		2017Q1		2017Q2		2017Q3		95% CI of 2017Q3
Coding practice 1: I10 a primary diagnosis															
Medical centers	333	(0%)	330	(−1%)	315	(−5%)	284	(−15%)	188	(− 44%)	105	(−68%)	85	(−74%)	− 73.8% to − 74.9%
Clinics	1448	(0%)	1465	(+ 1%)	1443	(0%)	1351	(−7%)	1221	(−16%)	1155	(−20%)	1111	(−23%)	−23.2% to − 23.3%
Coding practice 2: I10 in secondary diagnosis															
Medical centers	868	(0%)	955	(+ 10%)	916	(+ 6%)	873	(+ 1%)	811	(−7%)	756	(−13%)	734	(− 15%)	−15.4% to − 15.4%
Clinics	511	(0%)	533	(+ 4%)	532	(−2%)	500	(−2%)	447	(−13%)	430	(−16%)	417	(−18%)	−18.3% to −18.4%
Coding practice 3: I11 − I13 in primary diagnosis															
Medical centers	117	(0%)	134	(+ 14%)	131	(+ 12%)	129	(+ 10%)	140	(+ 19%)	192	(+ 64%)	203	(+ 73%)	72.7 to 73.8%
Clinics	254	(0%)	257	(+ 1%)	256	(+ 1%)	244	(−4%)	224	(−12%)	213	(−16%)	206	(−19%)	−18.9% to −19.2%
Coding practice 4: I11 − I13 in secondary diagnosis															
Medical centers	143	(0%)	159	(+ 12%)	154	(+ 8%)	151	(+ 6%)	152	(+ 6%)	153	(+ 7%)	152	(+ 7%)	6.7 to 6.8%
Clinics	99	(0%)	103	(+ 4%)	103	(+ 4%)	99	(0%)	89	(−10%)	86	(−13%)	83	(−17%)	−16.4% to −16.7%

*Abbreviations*: *ICD − 10 – CM* International Classification of Disease Tenth Revision Clinical Modification, *I10* Essential (primary) hypertension, *I11* Hypertensive heart disease, *I12* Hypertensive chronic kidney disease, *I13* Hypertensive heart and chronic kidney disease, *95% CI* 95% confidence intervals

^a^Using the number of visits of 2016Q1 as the base for % change

**Table 2:** Number (% change^a^) of outpatient visits (×1000) of four diabetes-related coding practices in outpatient claims by the same physician in tertiary medical centers (intervention group) and clinics (control group) from 2016Q1 to 2017Q3 in Taiwan (ICD-10-CM code E119 is the Outpatient Volume Control Program (OVCP) monitoring code and E110-E118 are non-OVCP monitoring codes)

	2016Q1		2016Q2		2016Q3		2016Q4		2017Q1		2017Q2		2017Q3		95% CI of 2017Q3
Coding practice 1: E119 in primary diagnosis															
Medical centers	270	(0%)	281	(+ 4%)	270	(0%)	252	(−7%)	186	(−31%)	94	(−65%)	72	(−73%)	− 72.8% to − 74.0%
Clinics	575	(0%)	609	(+ 6%)	610	(+ 6%)	566	(−2%)	511	(−11%)	492	(−15%)	475	(−17%)	−17.4% to − 17.5%
Coding practice 2: E119 in secondary diagnosis															
Medical centers	304	(0%)	318	(+ 5%)	305	(0%)	287	(−5%)	273	(−10%)	258	(−15%)	242	(−20%)	−20.3% to − 20.5%
Clinics	259	(0%)	275	(+ 6%)	272	(+ 5%)	250	(−3%)	219	(−15%)	208	(−20%)	198	(−24%)	− 23.5% to − 23.8%
Coding practice 3: E110 − E118 in primary diagnosis															
Medical centers	198	(0%)	222	(+ 13%)	219	(+ 11%)	208	(+ 5%)	234	(+ 18%)	285	(+ 44%)	289	(+ 46%)	46.0 to 46.6%
Clinics	139	(0%)	154	(+ 11%)	157	(+ 13%)	153	(+ 10%)	138	(−1%)	137	(−2%)	136	(−2%)	−2.2% to − 2.2%
Coding practice 4: E110 − E118 in secondary diagnosis															
Medical centers	112	(0%)	122	(+ 8%)	119	(+ 6%)	111	(−1%)	99	(−11%)	103	(−8%)	105	(−6%)	−5.8% to −5.9%
Clinics	41	(0%)	45	(+ 11%)	47	(+ 14%)	43	(+ 6%)	37	(−8%)	37	(−9%)	36	(−12%)	−11.5% to − 11.8%

*Abbreviations*: *ICD − 10 – CM* International Classification of Disease Tenth Revision Clinical Modification, *E119* Type 2 diabetes mellitus without complications, *E111 − E118* Type 2 diabetes mellitus with ketoacidosis, kidney, ophthalmic, neurological, circulatory, and other specific and unspecific complications, *95% CI* 95% confidence intervals

^a^Using the number of visits of 2016Q1 as the base for % change

A prominent decline in the number of visits with coding practice 1 (OVCP monitoring code as primary diagnosis) was noted for patients with hypertension (from 332,668 in 2016Q1 to 85,215 in 2017Q3 with a percentage change of − 74%) and patients with diabetes (from 269,928 in 2016Q1 to 71,776 in 2017Q3 with a percentage change of − 73%) in tertiary medical centers (intervention group). However, only a mild decline was noted in clinics with a percentage change of − 23% for patients with hypertension and − 17% for patients with diabetes.

By contrast, a drastic increase in coding practice 3 (non-OVCP monitoring code as primary diagnosis) was observed for patients with hypertension (from 117,221 in 2016Q1 to 203,056 in 2017Q3 with a percentage change of + 73%) and patients with diabetes (from 19,754 in 2016Q1 to 289,008 in 2017Q3 with a percentage change of + 46%) in tertiary medical centers (intervention group). However, a mild decline was observed in clinics with a percentage change of − 19% for patients with hypertension and − 2.2% for patients with diabetes.

Among the patients with hypertension, the percentage change in tertiary medical centers was similar to that in clinics: − 15% versus − 18% for coding practice 2 (OVCP monitoring code as secondary diagnosis) and 7% versus − 17% for coding practice 4 (non-OVCP monitoring code as secondary diagnosis). Among the patients with diabetes, the percentage change in tertiary medical centers was also similar to that in clinics: − 20% versus − 24% for coding practice 2 and − 6% versus − 12% for coding practice 4.

## Discussion

The findings of this study support three out of the four hypotheses we proposed (i.e., prominent decline in coding practice 1 [OVCP monitoring code recorded as primary diagnosis], drastic increase in coding practice 3 [non-OVCP monitoring codes recorded as primary diagnosis], and no noticeable change in coding practice 4 [non-OVCP monitoring codes recorded as secondary diagnoses] in tertiary medical centers). However, we did not observe any increase in coding practice 2 (OVCP monitoring code recorded as secondary diagnosis) in tertiary medical centers, which means that most physicians did not change the less severe codes from primary diagnosis to secondary diagnosis.

Several studies have addressed the physician code creep in outpatients after the establishment of a value-based program [7–12]. Using the Truven MarketScan data, one study indicated that the proportion of patients coded as having higher anesthesia risks increased from 11.6% in 2005 to 18.9% in 2013 for outpatient gastrointestinal endoscopy procedures [11]. The results, as suggested by the authors, could not be explained by the severity of the patients’ conditions nor be attributed to changes in the physician population. Furthermore, the changes in coding for anesthesia risks became more marked when the same physicians were examined over time [11].

A study used the Healthcare Cost and Utilization Project state databases also revealed that the proportion of outpatient percutaneous coronary interventions coded for acute indications increased prominently in New York (from 0.6% in 2010 to 8.3% in 2014), and the increase was due to a substantial rise in the number of coded unstable angina after the appropriate use criteria for coronary revascularization were released in 2009. The authors suggested the possibility of physicians increasingly classifying patients with stable chest pain as unstable angina in the outpatient setting [12].

Caskey et al. further suggested that there is a potential for financial disruption due to inaccurate mappings from ICD-9-CM to ICD-10-CM [20]. For example, ICD-9-CM 272.4 “other and unspecified hyperlipidemia” could be map to either E78.5 “hyperlipidemia unspecified” or E78.4 ‘Other hyperlipidemia.” As indicated by the authors, the decision of which code to select may seem arbitrary but the reimbursement may differ as one code may be a higher reimbursed service by a payer despite the fact there is no intuitive difference to the billing clinician [20].

Several inpatient data studies have indicated that hospitals could improve the pneumonia mortality and readmission rate by coding respiratory failure or severe sepsis as primary diagnosis; these patients would not be included in the calculation of the pneumonia mortality and readmission rate under the Hospital Readmission Reduction Program initiated by the Centers for Medicare and Medicaid Services [14–17].

Similarly, the Taiwan OVCP calculates the expected volume of outpatient visits only with 172 designated diagnoses as primary diagnosis. The findings of this study reveal that, at least in two of the designated diagnoses (ICD-10-CM code I10 and E119), the volume of visits declined prominently after the initiation of the OVCP mainly because of the decline in the recording of less severe ICD-10-CM code I10 or E119 as primary diagnosis and the compensatory increase in the recording of more severe ICD-10-CM code I11 − I13 or E110 − E118 as primary diagnosis.

The changes in coding practices may be considered as deceived behaviors. However, it might be suggested that these changes in coding practices are corrected behaviors. In Taiwan, every physician uses the electronic medical record (EMR) system in outpatient setting, and it is common for physicians to copy the records of a previous visit and modify the wordings for chief complains, present illness, findings of the physical examination, and prescriptions. Some physicians might not change the primary and secondary diagnosis of a previous visit. Furthermore, according to the ICD-10-CM Official Guidelines for Coding set by the Centers for Medicare and Medicaid Services and the National Center for Health Statistics, the level of details required for some ICD-10-CM codes might be different depend based on various circumstances in outpatient encounters, such as the observation stay, outpatient surgery, diagnostic only, and coexisting chronic diseases [21]. Most physicians might not be aware of these guidelines.

After the proposal of the OVCP, various tertiary medical centers designed pop out reminding notes in the EMR outpatient interface for when one of 172 designated diagnoses was recorded as primary diagnosis. The reminding notes will ask physicians: “Is this diagnosis the main reason of this visit?”, “Did the patient with hypertension have a heart or kidney disease? If yes, more appropriate codes such as ICD-10-CM codes I11−I13 could be used.”, or “Did the patient with diabetes have complications? If yes, more appropriate codes such as ICD-10-CM codes E110−E118 could be used.” That is, the initiation of the OVCP offers the opportunity to educate physicians to assign a more appropriate code as the primary diagnosis. Physicians will have more motivation to read the reminding notes because the codes recorded are related to the reimbursement. Further investigation is required to examine whether these changes were deceived or corrected coding behaviors.

One of the strengths of this study was that it was nationwide and population-based. The second strength was the use of a fix patient cohort design as suggested by Khera et al. [22] (i.e., patients visiting the same physician in the same hospital or clinic) to observe the changes of coding practices in each physician. Third, unlike previous studies that had focused on episodes and changes across years, this study confined to the same patients with chronic conditions (hypertension and diabetes), and the observation period was short (less than 2 years). Therefore, most of the changes in coding practices were more likely due to changes in the physicians’ coding behaviors rather than due to changes in the severity of patient conditions.

However, several limitations should be noted. First, approximately one seventh of the patients in tertiary medical centers (cohort 1_HT_TMC and cohort 3_DM_TMC) and one fourth of the patients in clinics (cohort 2_HT_Clin and cohort 4_DM_Clin) were lost during follow-up over the study period. We examined the coding practice of physician on the same patient. If the patient did not see the same physician in the following visits, which would result in the decreasing of certain coding practices and that is the reason why the percent change also decreased in control group (i.e., clinics). Second, data on the characteristics of patients and physicians were not available in this study; therefore, we could not further examine the difference in the changes of coding practices based on the characteristics of patients and physicians. Third, without gold standard evidence, we could not determine whether the replacement of non-OVCP monitoring codes as primary diagnosis corresponded to misguided or correct coding behaviors.

## Conclusions

Despite these limitations, this nationwide before-and-after observational study of four patient cohorts visiting the same physician in the same clinical setting suggests that the physician code creep (replacing less severe codes with more severe codes as primary diagnosis) in outpatient claims existed after the initiation of the OVCP. One implication of the findings of this study was that the validity of using outpatient diagnoses to calculate case-mix index or quality of care indicators should be threatened. Cautions should be noted while using outpatient diagnoses for value-added analysis. The education of appropriate outpatient coding for physicians will be relatively effective when proper coding is related to reimbursement.

## Data Availability

The datasets used and analysed during the current study are available from the corresponding author on reasonable request.

## Abbreviations

DRG: Diagnostic related group
EMR: Electronic medical record
ICD-10-CM: International Classification of Diseases Tenth Revision Clinical Modification
NHI: National Health Insurance
OVCP: Outpatient Volume Control Program
